# Decoding pain and mental health patterns in youth with chronic pain during COVID-19

**DOI:** 10.3389/fpain.2026.1760356

**Published:** 2026-04-29

**Authors:** Fareha Nishat, Sarah Brennenstuhl, Nicole E. MacKenzie, Sakib Tariq, Kathryn A. Birnie, Melanie Noel, Chitra Lalloo, Sabine Soltani, Jennifer N. Stinson

**Affiliations:** 1 Child Health Evaluative Science, The Hospital for Sick Children, Toronto, ON, Canada; 2 Lawrence Bloomberg Faculty of Nursing, University of Toronto, Toronto, ON, Canada; 3 School of Health and Medical Sciences, City St.George’s University of London, London, United Kingdom; 4 Department of Anesthesiology, Perioperative, and Pain Medicine, University of Calgary, Calgary, AL, Canada; 5 Department of Psychology, University of Calgary, Calgary, AL, Canada; 6 Alberta Children’s Hospital, Hotchkiss Brain Institute, Calgary, AL, Canada; 7 Institute of Health Policy, Management & Evaluation, University of Toronto, Toronto, ON, Canada

**Keywords:** chronic pain, clustering, latent profile analysis, mental health, youth

## Abstract

**Background:**

The COVID-19 pandemic led to major disruptions in healthcare services, resulting in increased pain and co-occurring mental health issues among youth with chronic pain. However, heterogeneity in these symptoms has not been explored, overlooking the possibility of distinct subgroups. This study aimed to: (1) identify latent profiles of youth based on pain and mental health symptoms; and (2) examine whether sociodemographic and pandemic-related factors were associated with profile membership.

**Methods:**

A cross-sectional study of 357 youth aged 8–18 years with chronic pain was conducted during the first three waves of the COVID-19 pandemic (August 2020–April 2021) in Canada. Data on pain, mental health (anxiety, depression, post-traumatic stress disorder, insomnia), and COVID-19 impact were collected using validated questionnaires, alongside self-reported age, sex, and ethnic identity. Latent profile analysis was used to identify unobserved subgroups based on pain and mental health symptoms, and the optimal model was selected based on statistical fit and clinical interpretability. Multinomial logistic regression examined associations between profile membership and covariates.

**Results:**

Five distinct subgroups of youth with chronic pain based on co-occurring mental health symptoms were found. The largest group (42.2%) reported sub-clinical mental health symptoms with the lowest pain, while 12.5% fell into a “sub-clinical mental health symptoms/high pain” group. About one-third (34.6%) were in a “moderate mental health symptoms/moderate pain” profile. Two smaller but clinically concerning groups emerged: “high mental health symptoms/high pain without clinical PTSD” (6.6%) and “high mental health symptoms/high pain” (4.1%). Multinomial logistic regression showed that older youth, female youth and higher perceived COVID-19 impact were associated with membership in nearly all elevated mental health symptom/pain profiles.

**Discussion:**

Youth with chronic pain showed heterogeneous experiences of mental health during the first three pandemic waves, shaped by sociodemographic and contextual factors. Findings underscore the need for person-centered approaches to pain management, particularly in periods of acute stress, that address both individual vulnerabilities and broader contextual stressors.

## 1 Introduction

Chronic pain in youth is increasingly common, with prevalence estimates suggesting 20% of youth experience pain lasting longer than three months (1). In Canada, this translates to over one million youth living with chronic pain (2). Chronic pain in youth is associated with reduced participation in social and recreational activities, difficulties in peer relationships, and increased healthcare utilization (3, 4). Importantly, pain often co-occurs with mental health concerns such as anxiety, depression, post-traumatic stress disorder (PTSD), and insomnia in this group (5–10). When present in isolation, these conditions are known to negatively impact health-related quality of life, and when they co-occur with pain, the impact is magnified, leading to compounded challenges in daily functioning and overall development (11–13). Although the cooccurrence of chronic pain and mental health concerns is well documented, less is known about how these symptoms cluster within youth and whether distinct subgroups with different levels of pain and mental health symptomology can be identified.

During the COVID-19 pandemic, strict public health measures, including school closures, physical distancing, restrictions on recreational and social activities, and reduced access to healthcare services, disrupted the lives of young people globally (14–16). Recent research suggests that youth were disproportionately affected by these changes, reporting increased levels of stress, loneliness, and uncertainty (17, 18). A large cross-sectional study of over 800 adolescents in Canada found that more than half of the youth reported symptoms of depression, anxiety, or PTSD, with family confinement stress emerging as a key predictor of poor mental health outcomes (19). Such findings highlight the disproportionate psychosocial burden faced by Canadian youth during this period.

Early in the pandemic, Meulders et al., proposed a theoretical model that suggested that extreme uncertainty, loss of control, and prolonged stress introduced by the pandemic may amplify pre-existing psychosocial vulnerabilities in individuals with chronic pain while diminishing resilience factors such as social support and positive affect (20). Evidence to support this model in youth with chronic pain found that pandemic-related factors such as social isolation, disruption of daily routines, limited access to in-person healthcare, and heightened family stress exacerbated both mental health and pain symptoms (21–24). For example, a national survey of Canadian paediatric pain clinics found widespread service disruptions during COVID-19, with most centres reporting increased symptom severity and psychological distress among youth (21, 25). While telehealth adaptations improved accessibility for some patients, clinicians qualitatively noted that isolation, family strain, and reduced physical activity contributed to worsening pain and mental health symptoms (e.g., anxiety, stress) (25). The pandemic was also not felt equally, with marginalized communities (e.g., women, lower socioeconomic status) taking the brunt of the impact, many of whom are also likely to experience pain (1, 26). Despite the evidence of increased psychological distress among youth broadly during the pandemic, and the increased vulnerability experienced by youth with chronic pain, limited research examined whether distinct subgroups of youth with chronic pain emerged based on patterns of co-occurring pain and mental health symptoms during this period. Understanding the intersection of pain and mental health, and which groups among youth with pain experience elevated symptoms during this period is therefore critical.

Existing research at the intersection of pain and mental health in youth predominately treats young people with pain as a homogenous group defined only by their age. This approach overlooks the heterogeneity in how symptoms present and cluster within individuals. Identifying subgroups may provide insights into which youth are most vulnerable to worsening mental health outcomes and pain, and who might benefit from tailored prevention or intervention strategies to mitigate this risk. Latent Profile Analysis (LPA), a person-centred statistical approach, offers a powerful tool for identifying subgroups of individuals based on patterns of responses across multiple variables (27). In youth with chronic pain, this approach is rare. To our knowledge, only one paediatric chronic pain study has used a person-centred approach to identify four psychosocial profiles characterized by varying levels of internalizing symptoms (i.e., depression and anxiety), fatigue, catastrophizing, and family responses to pain (28). However, this study did not explore the intersection of pain characteristics and mental health symptoms in these youth. In adults with chronic pain, three studies were identified: they reveal distinct clusters of pain and mental health symptoms (e.g., anxiety, depression, stress, substance use) typically presenting as a low symptom profile experiencing both low pain and mental health, a moderate group with low pain but elevated mental health symptoms and high symptom profile experiencing both elevated pain and mental health symptoms (29–31). In the context of the pandemic, the mechanism proposed by Meulders et al. suggest a non-uniform response from youth with chronic pain, however limited evidence has explored this heterogeneity (20).

The aim of this study was to explore the heterogeneity in pain and mental health symptoms among youth with chronic pain in Canada during the first three waves of the COVID-19 pandemic. Specifically, the objectives were to: (1) identify latent profiles of youth based on their pain and mental health symptoms; and (2) examine whether sociodemographic and pandemic factors were associated with membership to these profiles.

## 2 Methods

### 2.1 Study design and population

A cross-sectional survey was conducted during the first three waves of the COVID-19 pandemic in Canada (August 2020–April 2021). The present study represents a secondary analysis of a subsample from a larger study, this study focuses specifically on youth with chronic pain. The larger study aimed to understand the experiences of the pandemic on Canadian youth with chronic pain, their siblings and parents. Detailed information on the study design and procedures is available in a previously published open-access article (22).

Youth were eligible to complete the survey if they were: (1) aged 8–18 years; and (2) self-reported having chronic pain (e.g., persistent or recurrent pain lasting 3 months or longer).

Participants were recruited through two approaches: (1) web-based social media outreach; and (2) clinic-based recruitment. Social media advertisements were shared through Twitter/X, Instagram and TikTok by study partners: The Hospital for Sick Children (SickKids), Solutions for Kids in Pain (SKIP), Pain BC, Cassie & Friends, Rare Disease Foundation and The ILC Foundation to reach youth in the community. Clinic based recruitment involved sharing study materials with 11 paediatric tertiary care chronic pain clinics and two rehabilitation programs across Canada. Research ethics approval was obtained at the lead study site (The Hospital for Sick Children REB #1000070100) well as the other 10 sites across Canada. Recruitment material was available in both English and French and included a link to capture youth consent and the study questionnaires via REDCap.

### 2.2 Measures

The measures described below capture core domains of pediatric chronic pain, in alignment with the biopsychosocial model of pain and the PedIMMPACT recommendations for core outcome domains in pediatric pain research (32).

#### 2.2.1 Baseline characteristics

Sociodemographic measures assessed youth sex, age, and ethnic origin through an investigator developed self-report questionnaire. Due to the small cell counts across ethnicity categories, ethnic origin was dichotomized for the multinomial regression to white or racialized (i.e., youth belonging to Arab/West Asian, Indigenous, Black, Asian or Hispanic/Latin American). We acknowledge that race and ethnicity are distinct social constructs; however, this categorization reflects the structure of the original survey item and analytic constraints related to sample size.

#### 2.2.2 Pain intensity and interference

Youth self-reported their average pain intensity over the past 7 days on an 11-point numerical rating scale (NRS) from 0 (“no pain”) to 10 (“pain as bad as you can imagine”). This is a valid and reliable measure of pain intensity in youth (33–35). For youth populations categories of pain (mild, moderate and severe) are not standardized and show considerable variability, as such, cut points were not applied in this study. Rather, higher NRS scores were interpreted as more severe pain (36, 37). Pain interference was assessed using the 4-item Pain Interference subscale of the Patient-Reported Outcomes Measurement Information System (PROMIS; pediatric). It assess the extent that pain interfered with daily activities in the past 7 days, with higher scores indicating greater pain interference (38). For statistical analysis, raw scores were transformed into standardized T-scores.

#### 2.2.3 Depression and anxiety symptoms

Symptoms of depression and generalized anxiety were assessed using the Patient Health Questionnaire-2 (PHQ-2) (39) and the GAD-7 (40). The General Anxiety Disorder 7-Item (GAD-7) is widely used among youth has shown to be valid and reliable (40, 41). Youth rated how often they were bothered by symptoms of depression (PHQ-2) and anxiety (GAD-7) over the past two weeks using a 4-point Likert scale ranging from 0 (“not at all”) to 3 (“nearly every day”), with higher total scores indicating greater symptomology. Both scales have established clinical cutoffs that indicate the presence of clinically elevated symptoms (i.e., GAD-7 ≥ 8; PHQ-2 ≥ 3) (39, 40). Internal consistency was good in the current sample for the GAD-7 (*α* = .80), and satisfactory, although modest for the PHQ-2 (*α* = .64) (42).

#### 2.2.4 PTSD symptoms

The Child PTSD Symptom Scale for DSM-5 (CPSS-5) was used to assess symptoms of PTSD in the past month according to DSM-5 diagnostic criteria (43). The measure has excellent internal consistency, good test-retest reliability, and good convergent validity (43). A total severity score is obtained by summing 20 items assessing PTSD symptoms on a 5-point Likert scale from “not at all” to “6 or more times a week/almost always”, with higher scores indicating greater PTSD symptoms. Clinically elevated symptoms of PTSD are indicated by a total score of at least 31 (43). Internal consistency of the CPSS-5 was excellent in the current sample (*α* = .93).

#### 2.2.5 Insomnia symptoms

The Insomnia Severity Index (ISI) was used to measure the severity of subjective insomnia symptoms over the last two weeks on a 5-point Likert scale ranging from “none” to “very severe” (44). Total scores above 14 are considered clinically significant and indicative of insomnia. The ISI has been used in previous research on pediatric chronic pain and demonstrated to be a reliable and valid self-report measure of insomnia (45–47). The scale had good internal consistency in the current sample (*α* = .80).

#### 2.2.6 Health-related quality of life

The Pediatric Quality of Life Inventory-Adolescent Report (PedsQL 4.0) was used to assess health-related quality of life (HRQOL) across 4 domains (Physical, Emotional, Social, and School), with higher total scores signifying increased HRQOL (48). This measure has been shown to have acceptable reliability and validity among youth in pediatric health care settings (48). The PedsQL 4.0 had good internal consistency in the current sample (*α* = .87).

#### 2.2.7 COVID-19 impact

The 40-item COVID Impact Questionnaire (teen version) (38, 39) was used to assess financial/economic, health, social, occupational, and academic impacts of the COVID-19 pandemic and how closely connected individuals were to the pandemic, with a total higher score indicating greater impact (24). Originally developed to capture psychological stress during Hurricane Sandy and used in both adult and adolescents (49, 50), it was revised to capture the unique facet of the COVID-19 pandemic and shows good internal consistency (*α* = .76) in the current sample of youth with chronic pain.

### 2.3 Data analysis

The characteristics of the sample were summarized using descriptive statistics. Latent Profile Analysis was used to determine if there was heterogeneity in the sample that could be explained by grouping youth into unique profiles based on their mental health and pain symptoms and if so, the optimal number of profiles to best fit the data. The analysis was undertaken in Mplus (version 7) using the 3-step approach to identify the best fitting model and investigate associations between the latent profiles and observed covariates (51).

In Step 1, the optimal number of profiles was selected using seven measures of mental health and pain symptoms including: depression, anxiety, PTSD, insomnia, quality of life, average level of pain and pain interference. Models with increasing number of profiles were fitted to the data, beginning with a single profile model and including further profiles until group membership in any profile became too small (i.e., <5% of total). The best model was decided based on clinical and practical interpretability guided by a suite of standard model fit criteria: Akaike's Information Criterion (AIC), the Bayesian Information Criterion (BIC) and the sample size-adjusted BIC (lower Information Criteria values indicating better fit), a significant bootstrap likelihood ratio test (BLRT), a significant Lo-Mendell-Rubin Adjusted Likelihood Ratio Test and entropy value (higher values indicating better classification accuracy) (52).

In Step Two, once the best fitted model was determined, a variable representing the most likely profile was constructed based on the posterior distribution obtained from Step One for everyone in the sample (51). In Step Three, multinomial logistic regression was used to regress the most likely profile on the covariates, considering misclassification in the first step. The covariates included age, sex, race and COVID-19 impact.

The LPA models were estimated using maximum likelihood with robust standard errors, which uses all available data to decide on the best fitting model; however, in the last step, listwise deletion was used to address missing data on the covariates. This resulted in 2 missing cases (<.1%). With such a small proportion of missing data, imputation was not used. To avoid local maxima, the model was tested on a replicated solution using different starting values of the best maximum likelihood value and was found to converge (53). All tests were two-sided; *p* < .05 was used to evaluate statistical significance.

## 3 Results

### 3.1 Sample

Three-hundred and thirty-four youth with chronic pain completed consent to take part in the study, among which, 34 youth did not complete the survey. A further 7 were removed prior to analysis due to postal codes indicating the respondents were not in Canada, 28 survey responses were suspected to be repeats and 1 was a blank survey. The final sample included 357 youth with chronic pain, 172 (48%) were recruited from tertiary-level chronic pain clinics, 111 (31%) were recruited through social media and 74 (21%) were originally recruited as siblings of youth with chronic pain, however they endorsed having pain themselves. More than half the youth with chronic pain were female (58.9%), with an average age of 15.8 (SD = 1.9) years and identified as white (88.5%) ethnic origin. Further sociodemographic and pain characteristics are described in Table 1.

**Table 1:** Demographic and pain characteristics of youth with chronic pain.

Characteristics	Youth with chronic pain (*n*=357)
Demographic Factors	
Age, mean (SD)	15.8 (1.9)
Sex, *n* (%)	209 (58.9)
Ethnic origin, *n* (%)	
White	316 (88.5)
Arab/West Asian	5 (1.4)
Indigenous (First Nations, Métis, Inuit)	5 (1.4)
Black	31 (8.7)
Asian	14 (3.9)
Hispanic/Latin American	*n* < 5
Pain Characteristics	
Pain Intensity, mean (SD)	5.0 (1.8)
Pain Interference, mean (SD)	58.8 (5.2)
Pain duration, *n* (%)	
3–6 months	47 (13.2)
>6 months and <1 year	102 (28.6)
1–2 years	107 (30.0)
3–5 years	60 (16.8)
5–10 years	20 (5.6)
More than 10 years	14 (3.9)
Unknown	7 (2.0)
Pain Location*, *n* (%)	
Stomach	111 (31.1)
Head	162 (45.4)
Muscles and joints	140 (39.2)
Leg(s)	111 (31.1)
Chest	43 (12.0)
Other	42 (11.8)

* Participants could select more than one pain location; therefore, percentages do not sum to 100%.

### 3.2 Latent profile analysis

**Figure 1:**
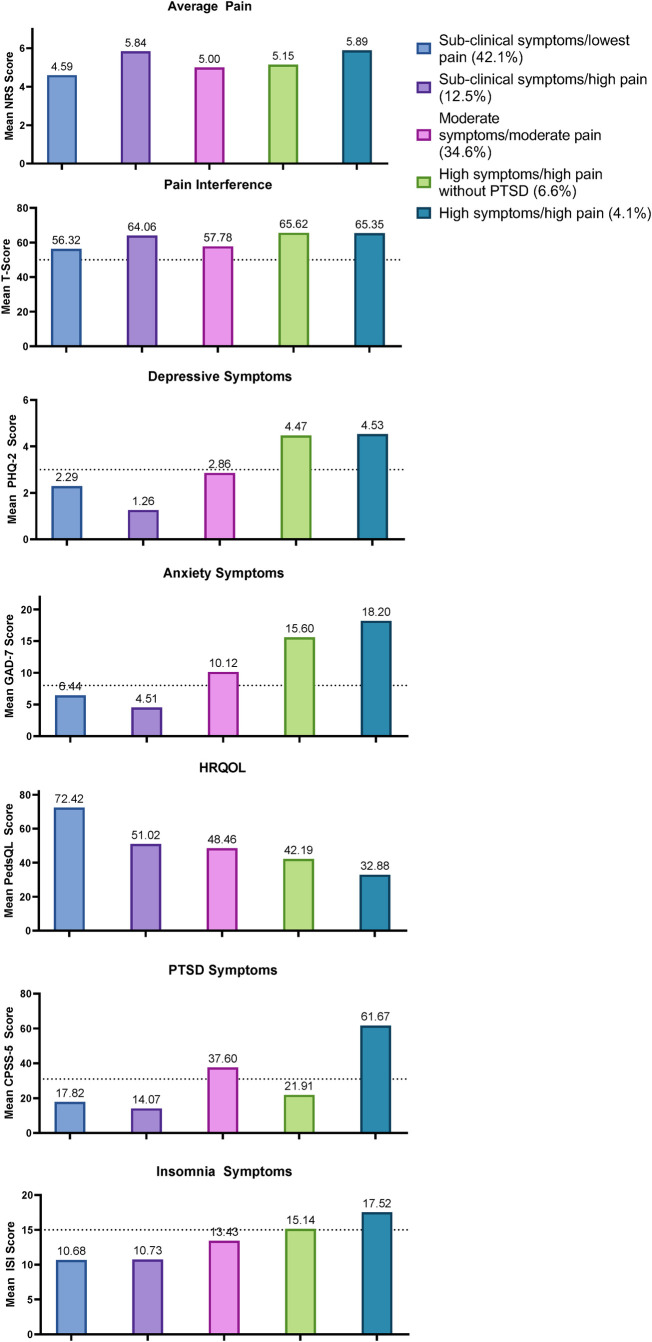
Mean pain and mental health symptoms across latent profiles. Dotted lines in the pain interference, depressive symptoms, anxiety symptoms, PTSD symptoms, and insomnia symptoms graphs represent established clinical cutoff scores; values above these lines suggest clinically significant symptom severity.

The best fitting model according to established criteria and clinical meaningfulness was a five-profile model. Table 2 provides a comparison of model fit criteria across models tested. The five-profile model had significant BLRT and adjusted-LMR values, a high entropy value of .90 indicating excellent classification accuracy and clear separation between profiles. While the information criteria values were not lowest, the number of groups became too numerous (and size of groups too small) in higher order models. Figure 1 presents the mean levels of pain and mental health symptoms found in each group.

**Table 2:** Comparison of LCA models with different numbers of classes according to selection criteria.

	Indicators of model fit					
Number of classes	BIC	AIC	Sample-size Adjusted BIC	Adjusted LMR test	BLRT	Entropy
1 class	14,826.104	14,771.815	14,781.689			
2 classes	14,501.119	14,415.809	14,431.325	0.2414	<.001	0.808
3 classes	14,311.858	14,195.779	14,216.685	0.0203	<.001	0.807
4 classes	14,341.084	14,193.73	14,220.531	0.2145	<.001	0.844
5 classes	14,275.184	14,096.808	14,129.25	0.0381	<.001	0.898
6 classes	14,180.999	13,971.601	14,009.686	0.0065	<.001	0.911

Note. BIC, Bayesian Information Criterion; AIC, Akaike's Information Criterion; LMR, Lo-Mendell-Rubin; BLRT, bootstrap likelihood ratio test.

Profile 1, characterized as the “sub-clinical mental health symptoms/lowest pain” group, representing 42.2% of the sample. This group has the lowest average pain level of 4.6, a pain interference t-score of 56.3, indicating that it is just above the population average of 50. Scores for depression, anxiety, insomnia and PTSD symptoms do not exceed clinical cutoffs, and mean QOL is highest among all groups at 72.4.

Profile 2, representing 12.5% of the sample, characterized as the “sub-clinical mental health symptoms/high pain” group. Average pain for this group is tied for the highest of all the groups at 5.8 and the mean t-score for pain interference is 64.1. However, mean scores for depression, anxiety, insomnia and PTSD symptoms remain below clinical cutoffs and mean QOL is second highest among the groups at 51.0.

Profile 3, representing 34.6% of the sample, characterized as the “moderate mental health symptoms/moderate pain” group. The average level of pain for this group is 5.0, and mean pain interference t-score is 57.8. Mean scores for anxiety and PTSD symptoms just exceed clinical cutoffs, while the mean score for depression and insomnia remain just below. Mean QOL falls mid-way among the groups at 48.5.

Profile 4, to be known as “high mental health symptoms/high pain without PTSD symptoms” group, representing 6.6% of the sample. Mean depression, insomnia and anxiety are all well above clinical cutoffs for this group; average pain is 5.2 and pain interference is among the highest of the groups, at 65.6. However, the mean PTSD symptom score of 21.9 is well below the clinical cutoff.

Finally, Profile 5, representing the smallest portion of the sample at 4.1%, is the “high mental health symptoms/high pain” group. This group is tied for the highest level of average pain (5.9) and pain interferences (*t*-score = 65.4), has the highest mean levels of depression, anxiety, insomnia and PTSD symptoms, all exceeding clinical cutoffs, and the lowest level of QOL at 32.9.

### 3.3 Covariates associated with latent profiles

The multinomial logistic regression establishing covariates of the profiles can be found in Table 3. The latent classes on the top row are compared to the latent classes on column.

**Table 3:** Multinomial logistic regression estimates [log-odds (standard error)] for covariates predicting latent profile membership.

Covariate	Latent profiles					
		Sub-clinical symptoms/lower pain	Sub-clinical symptoms/high pain	Moderate symptoms/moderate pain	High symptoms/high pain w/out PTSD	High symptoms/High pain
Age in years	vs. Sub-clinical symptoms/ lower pain	–	.38 (.12)**	.35 (.10)***	.18 (.11)	.40 (.14)**
	vs. Sub-clinical symptoms/high pain	−.38 (12)**	–	−.03 (.14)	−0.20 (.14)	.01 (.17)
	vs. Moderate Symptoms/Moderate Pain	−.35 (.10)***	.03 (.14)	–	−.17 (.24)	.05 (.14)
	vs. High Symptoms/High Pain w/out PTSD	−.18 (.11)	.20 (.14)	.17 (.14)	–	.22 (.16)
	vs.High Symptoms/High Pain	−.40 (.14)**	.01 (.17)	−.05 (.14)	−.22 (.16)	–
Female sex (v. male)	vs. Sub-clinical symptoms/ lower pain	–	.85 (.45)	.41 (.34)	1.96 (.73)**	20.5 (.45)***
	vs. Sub-clinical symptoms/high pain	−.85 (.45)	–	−.43 (.53)	1.11 (.82)	N/E
	vs. Moderate Symptoms/Moderate Pain	−.41 (.34)	.43 (.53)	–	1.54 (.77)*	20.09 (.53)***
	vs. High Symptoms/High Pain w/out PTSD	−1.96 (.73)**	−1.11 (.82)	−1.54 (.77)*	–	18.54 (.82)***
	vs.High Symptoms/High Pain	−20.5 (.45)***	N/E	−20.09 (.53)***	−18.54 (.82)***	–
Non-white Race (v. white)	vs. Sub-clinical symptoms/ lower pain	–	−.34 (1.06)	1.91 (.57)**	−.12 (.99)	−18.17 (1.06)***
	vs. Sub-clinical symptoms/high pain	.34 (1.06)	–	2.25 (1.10)*	.46 (1.13)	N/E
	vs. Moderate Symptoms/Moderate Pain	−1.91 (.57)**	−2.25 (1.10)*	–	−1.78 (1.09)	−20.07 (1.10)***
	vs. High Symptoms/High Pain w/out PTSD	−.12 (.99)	−.46 (1.13)	1.78 (1.09)	–	−18.29 (1.13)***
	vs.High Symptoms/High Pain	18.17 (1.06)***	N/E	20.07 (1.10)***	18.29 (1.13)***	–
Covid Impact (Total score)	vs. Sub-clinical symptoms/ lower pain	–	−.08 (.02)***	.11 (.02)***	−.01 (.03)	.08 (.05)
	vs. Sub-clinical symptoms/high pain	.08 (.02)***	–	.19 (.02)***	.07 (.03)**	.16 (.06)**
	vs. Moderate Symptoms/Moderate Pain	−.11 (.07)***	−.19 (.02)***	–	−.11 (.03)***	−.03 (.05)
	vs. High Symptoms/High Pain w/out PTSD	−.01 (.03)	−.07 (.03)**	.11 (.03)***	–	.08 (.06)
	vs. High Symptoms/High Pain	−.08 (.05)	−.16 (.06)**	.03 (.05)	−.08 (.06)	–

Values in the cell represent log-odds estimates from multinomial logistic regression models examining associations between covariates and latent profile membership. Each cell compares the latent profile listed in the column to the latent profile listed in the row. Positive estimates indicate that higher values of the covariate are associated with greater odds of membership in the column profile relative to the row profile, whereas negative estimates indicate greater odds of membership in the row profile relative to the column profile.

Bolded values represent statistically significant results at: *p < 0.05; **p < 0.01; ***p < 0.001.

*Age.* Older youth have significantly higher odds of being in the “sub-clinical mental health symptoms/high pain”, “moderate mental health symptoms/pain” and “high mental health symptoms/high pain” groups compared to the “sub-clinical mental health symptoms/lowest pain” group.

*Sex.* Females compared to males have higher odds of being in either “high mental health symptoms/high pain” or “high mental health symptoms/high pain without PTSD symptoms” groups compared to the “sub-clinical mental health symptom/lowest pain” group and the “moderate symptoms/moderate pain” group. Females also have higher odds of being in the “high mental health symptoms/high pain without PTSD symptoms” group than the “moderate mental health symptoms/moderate pain” group.

*Ethnicity.* Youth who identified as racialized compared to white youth have higher odds of being in the “moderate mental health symptoms/moderate pain” group than the “sub-clinical mental health symptoms/lowest pain” group or the “sub-clinical mental health symptoms/high pain” group; however, they had lower odds of being in the “high mental health symptoms/high pain” group than each of the “sub-clinical mental health symptoms/lowest pain”, “moderate mental health symptoms/moderate pain” and “high mental health symptoms/high pain without PTSD” groups.

*Covid Impact.* Those more impacted by COVID had higher odds of being in the “moderate mental health symptoms/moderate pain” group compared to either subclinical group. They were also more likely to be in the either of the high mental health symptom/pain groups than the “subclinical mental health symptoms/high pain” group. Finally, there were less likely to be in the “high mental health symptoms/high pain without PTSD” group than the “moderate mental health symptoms/moderate pain” group and less likely to be in the “sub-clinical mental health symptoms/high pain” group than the “sub-clinical mental health symptoms/lowest pain group”.

## 4 Discussion

### 4.1 Summary of results

This is one of the first studies to characterize distinct profiles of youth with chronic pain based on their co-occurring mental health symptoms during the first three waves of the COVID-19 pandemic, representing a novel contribution to the mental health and pain literature, highlighting these associations in the context of COVID-19. These findings reveal heterogeneity in how youth with chronic pain experienced mental health symptoms with demographic and contextual factors shaping risk for more severe profiles. A particularly clinically concerning group emerged which consisted of older youth, female youth and those with higher perceived COVID-19 impacts, who were shown to have more negative mental health outcomes in the context of higher pain. This study is the first to demonstrate these findings in the context of the COVID-19 pandemic, and highlights the importance of person-centred and contextual approaches that consider broader structural factors to clinical and community-based care and mental health support for youth with chronic pain, especially during periods of acute stress (54).

### 4.2 Comparison with previous literature

The heterogeneity of pain and mental health symptom profiles in youth with chronic pain is consistent with the existing literature using person-centred statistical approaches. In adult-focused literature, studies looking at mental health symptoms—typically including measures of anxiety, depression, stress and substance use—identified profiles broadly characterized by low pain-low psychological distress, moderate pain-moderate psychological distress and high pain-high psychological distress (29–31). Only one study has examined youth with chronic pain, identifying four psychosocial profiles characterized by varying levels of anxiety and depression, fatigue, catastrophizing, and caregiver protective responses to pain, although pain characteristics (intensity, interference, duration) were not included in the creation of these profiles (28). In the context of the COVID-19 pandemic, one study in adults with chronic pain also identified heterogeneity based on psychosocial distress, pain interference, and loneliness (55). However, to our knowledge, no studies have examined co-occurring pain and mental symptoms using person-centred statistical approaches in youth with chronic pain during the pandemic. Our findings provide empirical evidence for early theoretical models of the impact of COVID-19 on individuals with pain by demonstrating nuanced variability in youth responses to pandemic-related stressors, reflected in additional subgroups beyond those reported in adult and pre-pandemic youth studies (20). In addition to this important novelty, this study also provides confirmatory support for well-established patterns of stressors in the context of pain, demonstrating that under intense stress and uncertainty, known patterns of mental health and co-occurrence with pain may persist.

Several factors may explain why distinct profiles were identified in this study. One reason may be protective factors that likely reduced the disruption of the pandemic on some youth (23, 24, 56). The largest profile, consisting of youth with sub-clinical mental health symptoms and lowest pain may reflect fewer experiences of daily stressors and benefits from the increased flexibility that remote and asynchronous schooling offered (24, 56). Reduced academic and social pressures, more autonomy in managing their time, and decreased exposure to activities that could induce pain and/or anxiety may have acted as buffers for this group (23, 24, 56). At the family level, protective factors such as stable household income and housing, strong family functioning, and sustained social connection may have also contributed to their lower levels of pain and mental health symptoms (23, 57). Qualitative research from this time describes how some families living with chronic pain experienced the pandemic as an opportunity to strengthen family cohesion and mutual support, coming together to “build resilience” (56). Future work should examine which factors most strongly buffer against psychological distress in this group to inform family-centred supports.

However, it is important to note that even the profile conveying the fewest symptoms in comparison to other profiles, still reported moderate levels of pain. This aligns with the literature that youth experienced elevated pain and mental health symptoms during the pandemic (22, 23, 56, 58). Many of these studies also acknowledged that their study populations likely did not experience the most severe pandemic-related stressors, such as parental job loss, family income insecurity, housing instability, or the death of a family member (22, 23, 56, 58). Evidence from both the general population and among those with pre-existing chronic conditions indicates that these stressors were strongly associated with worse mental health outcomes (59–63). This is further supported by research following natural disasters (e.g., Hurricane Katrina), which found that greater physical and emotional losses predicted worse psychological distress (64, 65). This may explain the clinically vulnerable subgroup characterized by high pain and mental health symptoms that emerged, which were associated with greater perceived impacts of COVID-19. For these youth, the compounded effects of pre-existing vulnerabilities and severe pandemic-related disruptions may have amplified both pain and psychological distress.

Notably, two distinct high-pain profiles emerged: one characterized by co-occurring PTSD symptoms and another with elevated mental health symptoms with minimal PTSD symptoms. Consistent with research following natural disasters, PTSD symptoms in youth often arise in response to acute, traumatic exposures, including fear for one's safety or witnessing serious illness or death (64, 65). Given that data collection occurred during the first three waves of the pandemic, the sense of threat and the unprecedented nature of the pandemic may have contributed to the emergence of PTSD symptoms in some youth. However, this study was unable to determine the nature or timing of the traumatic event, as youth were not required to disclose such traumatic details for ethical reasons. Future research should explore whether these PTSD symptoms emerge from pre-existing trauma, pandemic-related structural adversities, or new traumatic events occurring during the study period. In contrast, the group with high mental health symptoms but with minimal PTSD symptoms may represent pre-existing vulnerabilities. Internalizing symptoms such as anxiety and depression were already more prevalent in youth with chronic pain prior to the pandemic, which may have been magnified under the uncertainty and isolation of the pandemic (5, 8). This suggests that even within those experiencing high pain, the source of psychological distress can differ, highlighting the need for tailored screening and interventions.

Interestingly, elevated pain could occur without clinical mental health symptoms, but the reverse was not true. In other words, profiles with clinical mental health symptoms always included elevated pain characteristics as well. This finding suggests that mental health difficulties may exacerbate pain experiences or reduce pain coping capacity. Prior research supports this relationship showing that depressive and anxious symptoms can heighten pain sensitivity and interfere with pain self-management (13, 66, 67). In the context of the pandemic, this dynamic may have been intensified by increased uncertainty, health-related fears, and social isolation.

This study also found that age, sex, ethnicity, and self-reported experiences of the pandemic shaped vulnerability. Older youth were more likely to fall into the more severe profiles, which aligns with developmental literature suggesting that this age period carries heightened sensitivity to stress and disruption (1, 68, 69). The initial waves of the pandemic were accompanied by major academic and social disruptions that may have compounded distress in this age group (15, 16, 70, 71). Similarly, the overrepresentation of female youth in severe profiles aligns with pain and mental health epidemiology, with females reporting higher rates of internalizing symptoms and pain sensitivity prior to the pandemic (1, 72). Patterns among racialized youth were mixed, although this may be a reflection of the sample being predominantly white, which limits our ability to draw meaningful conclusions. This warrants the need for more diverse and representative samples to understand how differences in the expression or reporting of pain and distress, or the presence of protective cultural and familial resilience mechanisms may buffer against the most severe outcomes (73–75). Finally, the strong association between higher perceived COVID-19 impact and membership in the high symptom profiles highlights the importance of contextual stress which may have exacerbated existing challenges for youth with chronic pain.

### 4.3 Clinical implications

This work holds important implications to inform clinical practice. These results suggest that higher pain and mental health symptoms appear to cluster in a substantial portion of youth, particularly in period of individual-, familial-, and community-level distress which aligns with the broader body of literature demonstrating the association between poorer mental health and chronic pain (5). Clinically speaking, this pattern of results bolsters the notion that youth who have experienced exposure to stressors during the COVID-19 pandemic are, in turn, more likely to demonstrate worsened mental health alongside greater levels of pain, as emerging work in this area has begun to demonstrate (20). Practically, this finding reinforces the imperative to conduct routine and thorough assessments of mental health in young people treated in chronic pain clinics or related contexts. Especially important to consider within these assessments are specific aspects of mental health, including insomnia, PTSD, mood, and anxiety, given their known impacts on chronic pain, and their potential to be exacerbated in stressful situations (12, 13, 46). Moreover, given the known impact on mental health and pain in the context of significant environmental and psychosocial stressors, as demonstrated through this work, it also calls attention to the critical nature of assessing the impacts of stressful life events and its potential contribution to pain and mental health. This requires a broad lens to not only understand the nature of stressors but the way in which systems respond to them, making social-ecological considerations within assessment and treatment very valuable. Indeed, the social systems that are known to influence pain and mental health may very well be impacted by phenomena such as the COVID-19 pandemic, making these essential systems to consider when assessing pain and mental health. With this broader psychosocial context accounted for, concerted and unified efforts are necessary to both effectively assess mental health in youth, identify appropriate interventions, and inform treatment goals, in light of stressors experienced by youth, whether it be the pandemic or other stressful life circumstances. Indeed, conducting thorough assessments of mental health that accounts for this creates an opportunity to engage youth in stepped care, a treatment approach whereby individuals are offered care that corresponds with their individual needs. This approach is shown to be effective in improving mental health symptomology (77). Such interventions have been developed to meet the unique needs of youth with chronic pain, such as Stinson and colleagues’ Power Over Pain Youth Portal, a virtual stepped care intervention to provide pain management and mental health supports to youth with chronic pain (78). Thorough assessments of pain alongside mental health have the potential to improve recommendations of resources and interventions through stepped care interventions such as this. Additionally, these profiles provide guidance on the identification of youth who may be at greatest risk of poorer health outcomes in the context of their pain and mental health. Indeed, assessment of these characteristics may help clinicians understand and stratify risk for interaction among mental health and pain symptoms. Youth who present with symptoms within the “high mental health symptoms/high pain” group profile characteristics may be regarded as having the greatest severity of symptoms and therefore greatest risk. As such, clinicians may wish to prioritize them for treatment or their presentations may warrant more intensve resources to support their pain and mental health. Future research should explore the health and mental health outcomes of these youth who experience these clusters of symptoms to provide clarity on the effectiveness of the nature of clinical interventions (e.g., timeliness of intervention, nature, intensity of treatment) and in turn, offer guidance on how best to clinically support these youth.

In addition to ensuring youth are provided with individualized care for mental health within the context of pain, the role of family resources, support, and family-level strain is important to consider given their known impacts on the mental health and pain symptoms explored in this study. Thus, it is critical to ensure that clinical interventions take a family-centred approach to care. It is well known that family and other contextual factors (e.g., family cohesion, affective responsiveness) can serve as key protective factors for youth with chronic pain and mental health concerns (79). Working in partnership with families to ensure they are provided with support to promote positive home environments and stability is necessary to support youth with making the gains described in the interventions above. Moreover, ensuring caregivers are engaged in care by providing them with adaptive coping techniques (e.g., managing protective behaviours) that can support their child may further increase the benefits for youth (80–82). To sufficiently address care at the individual and family level, it is critical to have representation of mental health professionals within multidisciplinary chronic pain teams. Clinical and health psychologists are particularly important to include, given their knowledge of evidence-based approaches to delivering mental health care within the context of chronic pain, while also being proficient in working with caregivers to create supportive home environments (83, 84). Having this expertise included within chronic pain teams is essential to provide wholistic care for youth with chronic pain.

### 4.4 Limitations

This study should be interpreted in the context of the following limitations. First, this was a cross-sectional study conducted during the first three waves of the COVID-19 pandemic in Canada. As such, results may not generalize to subsequent phases of the pandemic or other countries/regions, during which public health restrictions, healthcare access, and psychosocial stressors evolved. For example, recent research suggests increases in both chronic pain and psychological distress as the pandemic progressed (24). Moreover, it is recognized that cross-sectional research does not account for changes over time, and it is possible that profiles may cluster differently as the nature of the pandemic changes and different resources became available. This does, however, reinforce the conclusions drawn that consideration of the psychosocial context is a critical component to clinical assessment of these pain and mental health factors to ensure care is appropriately delivered. Additionally, the study population consisted predominantly of youth who identified as white from high income families, further limiting generalization. This finding is particularly important given that the pandemic disproportionately impacted marginalized and racialized communities, who may have experienced different barriers to care and psychosocial stressors (26). It is also important to note that ethnicity categories were collapsed due to small cell counts to allow for statistical comparison, this can obscure meaningful heterogeneity which may be present across ethnic groups and limits the interpretation of these findings. Indeed, future research and clinical work should assess and account for these factors to ensure relevance to the broader population, especially in the context of a diverse country such as Canada. Secondly, our recruitment approach used a convenience sample, which included social media advertisements to facilitate both rapid data collection (in line with the grant that supported this study) and to reach youth who may not be receiving access to tertiary chronic pain management. However, this approach included challenges such as automated or duplicate responses, and selection bias favoring families with greater digital access. Thirdly, given that the LPA is a data-driven approach specific to the sample used, these identified profiles are not definitive subgroups nor are they reflective of the general population due to the non-representative convenience sample (76). As such, future research should explore the validity of these profiles in independent samples. Furthermore, given that this study was cross-sectional, future longitudinal studies are needed to determine if these profiles persisted through the various phases of the pandemic. Again, this highlights the importance of careful and person-centred clinical assessments to understand the unique psychosocial factors contributing to each youth's clinical presentation in the context of pain and mental health. Fourth, as this was a secondary analysis, the surveys included were limited to the original study and were not selected specifically to address the aims of the current analysis. This may have limited the ability of LPA to detect nuanced subgroups since these depend on the number and quality of the indicators included (76). For example, there is potential for measurement error as the internal consistency of the PHQ-2 in this sample was modest, as a brief screener it may have not captured the full range of depressive symptoms. Moreover, all surveys relied on participant self-report; therefore, the identified profiles reflect youths' perceived pain and mental health symptoms rather than clinician-confirmed diagnoses. Future research incorporating multi-informant or clinician-rated assessments would strengthen confidence in these findings.

### 4.5 Conclusion

Our findings highlight the importance of a person-centred approach to chronic pain management in youth, recognizing the diverse ways in which pain and mental health symptoms cluster. The influence of pandemic-related factors on symptom severity points to the need for models of care that are responsive not only to individual needs but also to broader contextual stressors. However, to make informed and timely decisions that are tailored to the needs and context of each youth, routinely collected data is necessary. Together, these insights emphasize the value of flexible, integrated care models that can adapt both to the unique profiles of youth and to challenges that arise at the community or societal level.

## Data Availability

The raw data supporting the conclusions of this article will be made available by the authors, without undue reservation.

## COVIDChildPain Team includes

Samina Ali, Krista Baerg, Ardith Baerveldt, Marco Battaglia, Richard Bélanger, Myles Benayon, Fiona Campbell, Manon Choinière, Peter Cornish, Bruce D. Dick, Marie-Joëlle Doré-Bergeron, Sheri Findlay, Allen Finley, Pablo Ingelmo, Isabel Jordan, Lauren Kelly, Tieghan Killackey, Daniel Kopala-Sibley, Christine Lamontagne, Justina Marianayagam, Giulia Mesaroli, Vina Mohabir, Lori Montgomery, David Nicholas, Timothy Oberlander, Gabrielle Pagé, Raju Poolacherla, Patricia Poulin, Joshua Rash, Nivez Rasic, Mandeep Singh, Andrew Smith, Jaris Swidrovich, Peter Szatmari, Andrew Taylor, Joseph Charles Victor, Fotini Zachariades.
